# Tumor Metabolic Reprogramming by Adipokines as a Critical Driver of Obesity-Associated Cancer Progression

**DOI:** 10.3390/ijms22031444

**Published:** 2021-02-01

**Authors:** Duc-Vinh Pham, Pil-Hoon Park

**Affiliations:** 1 College of Pharmacy, Yeungnam University, Gyeongsan 38541, Korea; vinhpd@hup.edu.vn; 2 Research Institute of Cell Culture, Yeungnam University, Gyeongsan 38541, Korea

**Keywords:** adipokine, cancer metabolism, metabolic reprogramming, obesity

## Abstract

Adiposity is associated with an increased risk of various types of carcinoma. One of the plausible mechanisms underlying the tumor-promoting role of obesity is an aberrant secretion of adipokines, a group of hormones secreted from adipose tissue, which have exhibited both oncogenic and tumor-suppressing properties in an adipokine type- and context-dependent manner. Increasing evidence has indicated that these adipose tissue-derived hormones differentially modulate cancer cell-specific metabolism. Some adipokines, such as leptin, resistin, and visfatin, which are overproduced in obesity and widely implicated in different stages of cancer, promote cellular glucose and lipid metabolism. Conversely, adiponectin, an adipokine possessing potent anti-tumor activities, is linked to a more favorable metabolic phenotype. Adipokines may also play a pivotal role under the reciprocal regulation of metabolic rewiring of cancer cells in tumor microenvironment. Given the fact that metabolic reprogramming is one of the major hallmarks of cancer, understanding the modulatory effects of adipokines on alterations in cancer cell metabolism would provide insight into the crosstalk between obesity, adipokines, and tumorigenesis. In this review, we summarize recent insights into putative roles of adipokines as mediators of cellular metabolic rewiring in obesity-associated tumors, which plays a crucial role in determining the fate of tumor cells.

## 1. Introduction

Compelling evidence delineates that individuals with obesity have a higher risk of multiple malignancies, including endometrial cancer, hepatocellular carcinoma, colorectal cancer, esophageal adenocarcinoma, ovarian, and breast cancer [1,2]. Although extensive efforts have been devoted to unveiling the pathological connection between excess adiposity and cancer, the mechanisms by which obesity promotes these malignancies are not completely understood. Since aberrant production of adipokines, collectively referred to as hormones and cytokines derived from adipose tissues, has been recognized as one of the main pathological characteristics of obesity, there has been an increasing attention to their roles in the initiation, progression, and metastasis of various tumors [3,4]. A majority of adipokines, such as leptin, visfatin, and resistin, are overproduced during excess adiposity and have been postulated to be promoters of tumors [5,6]. On the contrary, adiponectin, which possesses the potent tumor-suppressing effects, is downregulated in obesity [7]. Therefore, an imbalance in adipokine production is considered one of the crucial factors for the development of obesity-linked cancers. However, the effect of each individual adipokine on tumorigenesis seems to be complicated and mediated through multiple mechanisms. For example, adipokines have been proposed to determine the fate of cancer cells via modulation of autophagy and tumor-promoting inflammation [8,9]. Furthermore, given the original role of adipokines as modulators of energy metabolism, adipokines may modulate tumor growth by regulating metabolic processes in tumor cells [10].

It has become increasingly clear that metabolic pathways in cancer cells are profoundly rewired to adapt to perturbations in a harsh tumor microenvironment. Since these metabolic adaptations seem to be an indispensable requirement for survival and exponential proliferation of tumor cells, understanding how cancer metabolism is remodeled and identifying factors, that drive metabolic remodeling, might open up new therapeutic opportunities [11]. In addition to cell-autonomous control by oncogenic signaling, cancer cells acquire metabolic adaptations to alterations in the tumor microenvironment, such as acidification, hypoxia, and poor nutrition [12]. Moreover, cancer cell metabolism is also regulated by interaction with adjacent stromal cells. Emerging evidence suggests that tumor-surrounding adipocytes promote the metabolic reprogramming in tumor cells through increased fatty acid availability and secretion of adipokines [13]. Instead, regulation of cancer cell metabolism by adipokines originating from distal adipose tissues can be mediated through an endocrinal mechanism [4]. In this review, we summarize the modulatory effects of adipokines on cancer cell-specific metabolism and highlight the crosstalk between other oncogenic signaling pathways and metabolic alterations driven by adipokines.

## 2. Adipokines and Cancer

Adipokines, produced and secreted exclusively or in large amounts by adipocytes or tissue-infiltrating immune cells, possess a wide range of biological activities and have been assumed to serve as a link between overweight- and obesity-associated complications. Unsurprisingly, many of them have been reported to play key roles in various malignancies. Among adipokines, leptin and adiponectin, which are predominantly produced from adipose tissue and represent true adipokines, have received the most attention as modulators of tumorigenesis. In addition, there is a growing appreciation of the importance of newer adipokines, such as resistin, visfatin, and apelin, in obesity-linked cancers.

### 2.1. Role of Leptin in Cancer

Leptin, considered the first identified adipokine, is produced in proportion to fat mass. Enhanced expression of leptin and its transmembrane receptors (Ob-R) have been observed in malignant tissues of the breast, lung, colon, uterus, liver, and ovary [14]. Furthermore, compelling evidence shows that leptin signaling play an indispensable role in obesity-promoted tumorigenesis. Indeed, while elevated body weight is believed to promote tumorigenesis, obese mice with deficits in the expression of leptin or leptin receptors failed to develop mammary tumors in a mouse mammary tumor virus enhancer/promoter-transforming growth factor alpha transgenic mouse model [15,16]. Likewise, obese rats bearing a leptin receptor missense mutation become less sensitive to chemical-induced breast carcinogenesis than lean wide-type rats [17]. Leptin has been reported to involve all the developmental stages of tumors, including initiation, growth, angiogenesis, and metastasis. Mechanistically, leptin activates important survival and proliferative signaling pathways such as Janus kinase (JAK)/signal transducer and activator of transcription (STAT), phosphatidylinositol 3-kinase (PI3K)/Akt, and mitogen-activated protein kinase kinase (MEK)/extracellular signal-regulated kinase 1/2 (ERK1/2) [5]. Moreover, up-regulation of autophagic process and inflammasomes activity [18,19], stimulation of cancer stem cell self-renewal [20], and promotion of epithelial-mesenchymal transition [21] have recently been suggested to be partly responsible for the tumor-promoting effects of leptin.

### 2.2. Role of Adiponectin in Cancer

Adiponectin, the most abundant adipokine in circulation, is one of the few adipokines whose production is downregulated during obesity. In line with the promoting role of excess adiposity in carcinogenesis, a decrease in circulating levels of adiponectin and/or adiponectin/leptin ratio is associated with an increased risk of obesity-induced cancers, including breast, endometrial, prostate, hepatic, and colorectal cancers [7]. Anti-cancer effects of adiponectin are mostly mediated via the membrane receptors AdipoR1/2. Binding of adiponectin with AdipoR1/2 causes activation of signaling pathways that lead to induction of apoptosis and cell cycle arrest through phosphorylation of AMP-activated protein kinase (AMPK) and downregulation of survival and proliferative signals, such as mitogen-activated protein kinase (MAPK), JAK/STAT, mammalian target of rapamycin (mTOR), and Wnt/β-catenin [7,22]. Adiponectin has been well recognized as a potent inducer of autophagy, which may contribute to cytotoxic effects in breast cancer cells [23]. In addition, modulation of estrogen receptor (ER) stress and NLRP3 inflammasomes are also involved in determination of cancer cell fate by adiponectin [24]. It is noteworthy that adiponectin antagonizes the oncogenic actions of leptin, supporting the epidemiological evidence that aberrant secretion of adipokines would results in development of cancer. In particular, a decreased adiponectin/leptin ratio predisposes patients to cancer development [25,26]. While adiponectin was originally considered to inhibit tumor formation and growth, several studies have also suggested that adiponectin may promote tumor progression. For examples, adiponectin has been shown to exhibit pro-angiogenic actions in mouse models of breast tumor, at least in part via T-cadherin, another membrane receptor of adiponectin [27,28]. Likewise, adiponectin induces cell migration and invasion in breast cancer cells [29,30]. However, adiponectin has also been shown to inhibit these malignant behaviors of cancer cell [31,32]. These contrasting findings imply that adiponectin might play multifaceted roles in cancer and its role would be determined in a context-dependent manner.

### 2.3. Effects of Other Adipokines in Cancer

Visfatin, also known as pre-B-cell colony-enhancing factor (PBEF) or nicotinamide phosphoribosyltransferase (NAMPT), is an adipokine that has recently emerged as a modulator of tumorigenesis. Visfatin was found overexpressed in tumor tissues of the breast, pancreas, and kidney compared to non-malignant adjacent tissues [33]. Furthermore, a high serum level of visfatin corresponds to poor prognosis in patients with breast cancer, gastric cancer, hepatocellular carcinoma, and urothelial carcinoma [34]. In addition, preclinical investigations have consistently revealed that visfatin supports the growth, angiogenesis, migration, and invasion of cancer cells [35,36,37]. While a specific receptor for visfatin has not yet been identified, its tumor-promoting effects are dependent on NAMPT enzymatic activity [38] or mediated via transactivation of signaling pathways, such as the PI3K/Akt, MAPK, and STAT3 signaling pathways [33,37].

Similar to leptin and visfatin, other adipokines overexpressed in obesity, such as resistin, apelin, and chemerin, also possess some oncogenic functions [39,40,41]. Yet, the detailed mechanisms by which these adipokines modulate cancer cell survival, growth, migration, and metastasis remain to be elucidated. Moreover, adipose tissues secret a number of other cytokines, including interleukin-6 (IL-6), tumor necrosis factor α (TNFα), and monocyte-chemoattractant protein-1 (MCP-1), which may also contribute to obesity-promoted cancer initiation and progression [13].

## 3. Effects of Adipokines on Cancer Metabolism

Metabolic reprogramming has long been considered a hallmark of malignancies. It was first observed that cancer cells appear to be dependent on glycolysis rather than mitochondrial oxidative phosphorylation (OXPHOS), even under aerobic conditions, subsequently known as the Warburg effect [42]. Since this observation, numerous advances in our understanding of how metabolic pathways are rewired in tumor cells have been made over the past decades. It is now clear that metabolic remodeling is not only limited to glycolysis but also other common metabolic pathways, including oxidative phosphorylation, lipid metabolism, and metabolism of amino acids and nucleotides. According to Thompson et al., cancer-related metabolic phenotypes can be organized into six hallmarks, including (1) dysregulation of glucose and amino acid uptake; (2) use of opportunistic modes of nutrient acquisition; (3) use of glycolysis/ tricarboxylic acid cycle (TCA) cycle as a supplier of substrates for biosynthesis and nicotinamide adenine dinucleotide phosphate (NADPH) production; (4) increased need for nitrogen; (5) alterations in gene regulation in response to metabolites; (6) metabolic interactions with the microenvironment [43]. Together, these rewired metabolic features fuel the cancer cell growth by fulfilling a high need for energy and biosynthesis of macromolecules. Furthermore, metabolic plasticity supports the survival of cancer cells under unfavorable conditions, such as oxidative stress, acidification, hypoxia, and nutrient depletion, which might be indispensable for tumor progression and metastasis [12]. Recent insights into cell-intrinsic and cell-extrinsic factors that promote metabolic alterations in cancer cells suggest that cancer metabolism is heterogeneous and driven by oncogenes, as well as components of the tumor microenvironment. In fact, stromal cells in the tumor microenvironment, such as fibroblasts, endothelial cells, adipocytes, and immune cells, may promote metabolism of tumor cells either by providing energy substrates or by secreting signaling molecules [11]. For example, cancer cells can mobilize fatty acids from adipocytes surrounding tumor [44] and cholesterol from tumor-associated macrophages to satisfy their metabolic demands [45]. Given the metabolic plasticity as a critical survival mechanism of cancer cell, targeting the drivers of cancer metabolism might be a promising strategy for cancer therapy. Recent reports on the modulatory roles of adipokines in cancer metabolism and the effects of adipokines-induced metabolic alterations on cancer cell fate will be discussed in this section.

### 3.1. Effects of Adipokines on Glycolysis and the Warburg Effect

The Warburg effect, a phenomenon characterized by a preference of aerobic glycolysis for glucose metabolism, is the first identified metabolic phenotype of cancer cells. While it was initially assumed that impaired respiration due to dysfunctional mitochondria underlines this phenomenon, further investigations reveal that the Warburg effect is a programmed process driven by a variety of oncogenes. For example, overexpression of glucose transporters (GLUTs) results in increase in uptake of glucose and upregulation of glycolytic flux as a consequence [46,47]. Furthermore, beyond the initial misinterpretation that cancer cells merely harbor glycolysis as a less efficient way for ATP production, intermediates generated during glycolysis can be diverted into branching pathways that allow coupling between glycolysis and other metabolic pathways, such as pentose phosphate, hexosamine, phospholipid, and amino acid biosynthesis [43]. Therefore, elevated glycolytic flux offers biosynthetic and proliferative advantages to malignant cells.

A large body of evidence has suggested that leptin enhances the Warburg effect via various mechanisms. First, leptin fosters uptake of glucose through upregulation of GLUT-1, which has been consistently observed in both malignant and non-malignant cells [48,49,50]. Second, leptin promotes glycolysis by modulating the expression and/or activity of rate-limiting enzymes within glycolytic pathways, including hexokinase, pyruvate kinase M2 (PKM2), and lactate dehydrogenase A (LDHA) [49,51]. Notably, leptin modulation of PKM2, an enzyme that converts phosphoenolpyruvate to pyruvate (the final product of glycolysis) in breast cancer cells, cholangiocarcinoma, and breast adipose stromal cells, has been found to be essential for stimulation of the epithelial-mesenchymal transition (EMT), cell migration, and invasion by this adipokine [52,53,54]. In line with these findings, increased uptake of glucose by leptin is coupled with a high flux of glycolysis and lactate production [49,55,56]. Recent evidence also indicates that leptin-stimulated breast cancer cells prefer using glucose for biosynthesis rather than energy production [57]. Moreover, the optimal growth of leptin-stimulated breast cancer cells has been observed in high glucose culture conditions [58]. Mechanistically, activation of PI3K/Akt seems to play a critical role in leptin-driven glucose metabolism in cancer cells. Indeed, while leptin has been well known as a prominent activator of PI3K/Akt, this signaling pathway is postulated to be a master regulator of glucose uptake and glycolysis [59,60]. Blockage of PI3K by a selective pharmacological inhibitor (LY294002) led to abrogation of alterations in glucose metabolism, as well as malignant behaviors promoted by leptin in cancer cells [49,52]. In addition, stabilization of HIF1α might also contribute to leptin regulation of glycolysis. This notion is supported by the evidence showing that HIF1α is a critical driver of the Warburg effect [61] and that leptin stabilizes HIF1α in cancer cells [53,62]. These studies also suggest that leptin induces the stabilization of HIF1α through suppression of p53, which results in HIF1α-dependent transcriptional activation of glycolytic enzymes, such as PKM2, hexokinase, and LDHA.

In addition to leptin, adiponectin also has exhibited well-recognized glucose metabolic effects. Under physiological conditions, adiponectin increases insulin signaling, which leads to improvement in glucose uptake and utilization for the maintenance of energy homeostasis [63]. Adiponectin-stimulated glucose uptake is mediated by enhanced GLUT-4 translocation in skeletal muscle cells [64]. Unfortunately, the effect of adiponectin on glucose uptake has not yet been confirmed in cancer cells. Given the difference in the metabolic phenotypes between tumor cells and non-malignant cells, whether adiponectin promotes glucose uptake in cancer cells and its possible role in adiponectin modulation of tumor growth remain to be elucidated. Indeed, in contrast to myocytes that mainly express GLUT-4, most cancer cells overexpress GLUT-1 [46]. Notably, a recent study has shown that adiponectin inhibits the expression of GLUT-1 in placental cells [65]. Regardless of how glucose import is affected by adiponectin, current evidence appears to support the concept that adiponectin stimulates oxidative phosphorylation of glucose, but represses glycolysis and lactate production, which is opposite to the Warburg effect [65,66,67]. To date, many of the biological actions of adiponectin on metabolism have been shown to be mediated via AMPK activation [68]. In addition, phosphorylation of AMPK by adiponectin was demonstrated to be a critical event underlying its anti-cancer effects [69]. Intriguingly, it has been recently documented that AMPK is a negative regulator of the Warburg effect. In particular, genetic ablation of AMPK led to a metabolic shift in aerobic glycolysis through normoxic stabilization of HIF1α, and as a result, promoted tumor progression [70]. Therefore, adiponectin signaling might play a role as a restrainer of the Warburg effect in cancer cells. Further insights into adiponectin modulation of glucose metabolism may lead to a better understanding of how adiponectin suppresses carcinogenesis.

**Figure 1:**
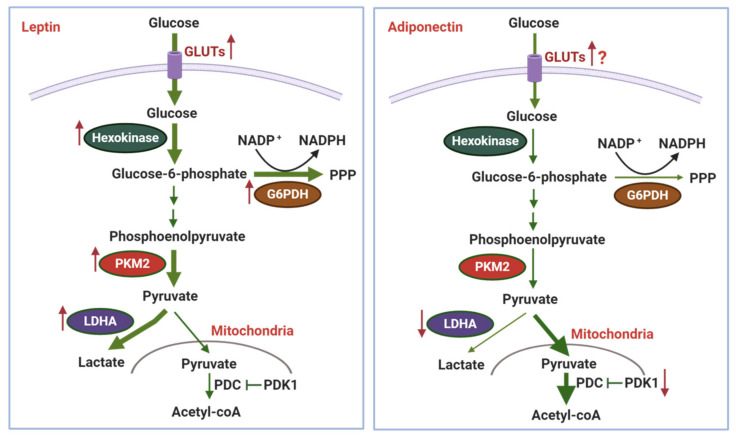
The modulatory effects of leptin and adiponectin on glucose metabolism in cancer cells. Glucose metabolism is profoundly remodeled in tumor cells, characterized by upregulation of glucose uptake and glycolytic pathway. Leptin promotes glycolysis by elevating expression and membrane translocation of glucose transporters (GLUTs), as well as increased expression of key enzymes in this pathway, including hexokinase, the M2 isoform of pyruvate kinase (PKM2), and lactate dehydrogenase A (LDHA). In addition, leptin increases expression of glucose-6-phosphate dehydrogenase (G6PDH), leading to upregulation of pentose phosphate pathway (PPP) for generating nicotinamide adenine dinucleotide phosphate (NADPH), pentose, and ribose-5-phosphate. In contrast, adiponectin appears to counteract the Warburg effect. Through activation of AMP-activated protein kinase (AMPK), it is assumed that adiponectin downregulates LDHA to inhibit the production of lactate from pyruvate but also reduces expression of pyruvate dehydrogenase kinase 1 (PDK1), a suppressor of pyruvate dehydrogenase complex (PDC), to increase the conversion of pyruvate to acetyl-CoA. Enlarged arrows denote the metabolic pathways activated by leptin/adiponectin.

In summary, accumulating evidence delineates that leptin and adiponectin show the opposite effects on glycolytic flux. Leptin promotes the utilization of glucose for glycolysis and biosynthesis, whereas adiponectin reverses, at least in part, the Warburg effect in cancer cells (Figure 1). However, as mentioned above, heterogeneity and complexity of cancer metabolism make it difficult to draw a general conclusion on the contribution of glucose metabolic alterations to the modulation of tumor growth by these adipokines. On the other hand, little has been known about the role of other adipokines in glucose metabolism in tumor cells. Limited data suggest a promoting role of visfatin in glycolysis. Similar to leptin, visfatin activates PI3K/Akt signaling and increases glucose uptake by enhancing the expression and membrane translocation of GLUT-1 [71,72]. Furthermore, since NAD^+^ is a mandatory coenzyme for oxidation of glyceraldehyde 3-phosphate in the glycolytic pathway, phosphoribosyltransferase activity of visfatin, which plays a central role in the regulation of NAD^+^ homeostasis, may boost glycolysis in cancer cells. In fact, inhibition of enzymatic activity of visfatin reduces cellular NAD^+^ pool, leading to the blockage of glycolysis [73]. Taking into consideration overexpression of visfatin in numerous cancer cells, visfatin-catalyzed NAD^+^ generation might be an important source of NAD^+^ to support the Warburg effect.

### 3.2. Effects of Adipokines on Mitochondrial Metabolism

Although a switch from oxidative phosphorylation (OXPHOS) to glycolysis is commonly observed in cancer cells, it is being increasingly appreciated that mitochondrial metabolism critically contributes to cancer cell life and proliferation [74]. In fact, even when glycolysis can provide enough ATP for cancer cells under in vitro optimal growth conditions, OXPHOS is essentially required in rapidly proliferating malignant cells [75]. Indeed, while glucose is preferred for aerobic glycolysis and anabolic metabolism, metabolic plasticity allows tumor cells to use other energy substrates, particularly fatty acid and glutamine, depending on tumor microenvironmental conditions [76]. In addition to functioning as a main source of ATP, mitochondria play a key role in cellular metabolism by providing intermediates for anabolism, regulating redox balance, calcium homeostasis, and cell death [77].

Accumulating evidence suggests that leptin elicits multifaceted effects on biogenesis, dynamics, and metabolic functions of mitochondria, which in turn underlies modulation of cancer metabolism and cancer cell fate. A recent study reported that leptin elevates oxygen consumption rate (OCR) and increases the dependence on mitochondrial OXPHOS for ATP production in breast cancer cells [57]. In addition, leptin upregulates genes related to mitochondrial biogenesis and dynamics, while total mass of mitochondria remains to be unchanged [78]. Along with the evidence indicating the mitophagy-inducing effect of leptin, these findings suggest that leptin confers growth and survival advantages to breast cancer cells by improving mitochondrial quality and function. Likewise, leptin was found to promote OXPHOS and ATP production in colon cancer cells [79]. In contrast, other investigations revealed that leptin suppresses OXPHOS in cancer cells. In the polyomavirus middle-T oncogene mouse model driven by the mouse mammary tumor virus promoter (MMTV-PyVT), the lack of peripheral leptin receptors led to a metabolic phenotype that was less reliant on aerobic glycolysis, but displayed enhanced OXPHOS capacity, which was involved in the reduction of tumor progression and metastasis [80]. It was also reported that colon cancer cells exposed to conditioned media from cultured human adipose tissue or exogenous leptin results in a drastic decrease in mitochondrial respiration and expression of mitochondrial proteins [81]. Interestingly, while previous studies have failed to draw a consistent conclusion on the effect of leptin on mitochondrial OXPHOS, most of the aforementioned changes in mitochondrial metabolism promote malignant behaviors in leptin-stimulated cancer cells. Therefore, we can put forward a hypothesis that leptin confers metabolic plasticity to cancer cells that facilitates survival and proliferation under different growth conditions. This notion is supported by recent evidence showing that leptin modulates mitochondrial homeostasis during metabolic stress. In prostate cancer cells cultured in hypoxia, a condition leading to impaired mitochondrial respiration, leptin stimulates mitochondrial biogenesis, stabilization of mitochondrial membrane potential, and elevated uncoupled respiration through HIF-1α-dependent upregulation of uncoupling protein 2 (UCP2) [62]. These metabolic alterations crucially contribute to maintenance of cancer progression in hypoxic environments. It is also worthy to note that leptin appears to promote fatty acid β-oxidation (FAO)-dependent energy production, since leptin was found to induce FAO in multiple cancer-related contexts. The effects of leptin on FAO as well as other aspects of lipid metabolism will be discussed in detail in Section 3.3.

The role of other adipokines in mitochondrial metabolism in cancer cells is not well defined. Although adiponectin is a physiological stimulator of mitochondrial biogenesis and respiration [82], it is unclear how adiponectin affects these processes in malignant cells. Likewise, the role of mitochondrial metabolism in oncogenic effects of other adipokines, including visfatin and resistin, remains to be investigated.

### 3.3. Effects of Adipokines on Lipid Metabolism

Lipid metabolism represents a relatively new area in cancer metabolism but has recently received increasing attention for its role in carcinogenesis. Compelling evidence demonstrates that lipid metabolism is profoundly remodeled in cancer cells. One of the key features of rewired lipid metabolism is increased de novo fatty acid synthesis, regardless of the availability of exogenous lipids through overexpression of lipogenesis-related genes [83]. This metabolic change is thought to provide cancer cells with a constant supply of fatty acids for energy production, membrane biogenesis, and post-transcriptional modification of proteins. However, it has subsequently been found that cancer cells can overcome pharmacological inhibition of fatty acid synthesis by elevated uptake of exogenous fatty acids [84]. In addition, cancer cells can store redundant lipids as lipid droplets, which further offers metabolic plasticity to malignant cells [85]. Another important alteration in cancer-linked lipid metabolism is increased oxidative degradation of fatty acids (β-oxidation) that generates a large number of acetyl-coA molecules, an intermediate molecule playing a central role in cellular metabolism [86]. Recently, dysregulation of cholesterol metabolism has been also linked to malignant behaviors of tumor cells [87].

**Figure 2:**
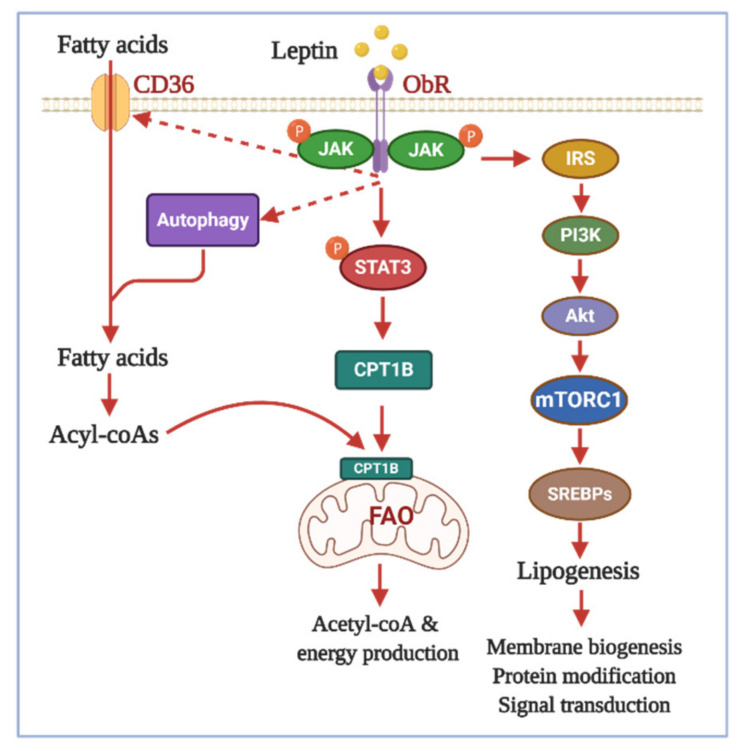
Leptin-promoted lipid metabolic reprogramming in cancer cells. Leptin modulates cancer cell specific lipid metabolism through concerted actions on biosynthesis, degradation, and uptake of fatty acids. On the one hand, leptin stimulates de novo fatty lipid biosynthesis through activation of SREBPs in a PI3K/Akt/mTORC1-dependent mechanism. Activation of lipogenesis by leptin offers proliferative facilitates to tumor cells via promotion of membrane biogenesis, post-transcriptional modulation of protein (e.g., palmitoylation and acetylation), and lipid-based signal transduction. On the other, leptin increases fatty acid β-oxidation (FAO) to meet a high-energy demand for rapid growth of tumor cells. To do so, leptin transcriptionally activates CPT1B via upregulation of the JAK/STAT3 signaling pathway to promote transportation of fatty acids into mitochondria. In addition, autophagy induction and elevated CD36-mediated uptake of exogenous fatty acids further increases availability of free fatty acids to fuel FAO. Together, the alterations in lipid metabolism have been demonstrated to critically contribute to oncogenic effects of leptin. Akt: protein kinase B, CD36: cluster of differentiation 36, CPT1B: the carnitine palmitoyl transferase 1B; IRS: the insulin receptor substrate, JAK: Janus kinase, mTORC1: mammalian target of rapamycin complex 1, ObR: the leptin receptor, PI3K: phosphatidylinositol 3-kinase, SREBPs: sterol regulatory element-binding proteins, STAT3: signal transducer and activator of transcription 3.

Among various adipokines, leptin and adiponectin have been documented to modulate lipid metabolism in cancer cells in a complicated manner. These adipokines differentially remodel cellular lipid metabolism by multifactorial regulation of biosynthesis, acquisition, and utilization of lipids. Although leptin possesses the physiological functions as an inhibitor of lipogenesis, emerging evidence suggests a promoting role of leptin in de novo biosynthesis of fatty acids in cancer-related contexts. Indeed, leptin enhances lipid droplet formation in intestinal epithelial cells, accompanied by high expression of markers of inflammation and malignant transformation [88]. Therefore, excess accumulation of lipid droplets by leptin is thought to contribute to the obesity-linked enhanced susceptibility to colon carcinoma. Increased reservation of intracellular lipids was also observed in leptin-treated breast cancer cells as a direct consequence of upregulation of the genes related with fatty acid synthesis. For example, leptin induces the expression of sterol regulatory element-binding proteins (SREBPs), including SREBP-1 and SREBP-2, master regulators of fatty acid and cholesterol synthesis, respectively, in breast cancer cells [89,90], which is opposite to the effects observed in non-transformed cells [91,92], suggesting that induction of SREBPs is a cancer cell-specific modulation of lipid metabolism by leptin. Notably, blockage of SREBPs abolishes leptin-stimulated cancer cell proliferation, migration, and invasion, indicating the critical role of SREBPs-dependent lipogenesis in oncogenic actions of leptin. In addition to upregulation of lipogenesis, leptin elevates the cellular lipid pool by increasing uptake of exogenous fatty acids. This effect is mediated via stimulation of expression and membrane translocation of cluster of differentiation 36 (CD36), a scavenger receptor that plays a critical role in fatty acid import [57,93]. Intriguingly, CD36 overexpression has been found to be associated with a more malignant phenotype of breast cancer cells [94,95]. Taken together, these findings support the notion that leptin drives fatty acid metabolic rewiring toward increased abundance of intracellular lipids, which in turn promotes cancer progression. It is also interesting to note that while lipogenesis and lipolysis are opposite processes that rarely coexist under physiological conditions, leptin stimulates lipid degradation through FAO [79,94]. Importantly, leptin-stimulated FAO has been implicated in many different aspects of tumorigenesis. First, since FAO represents a model of high-efficiency energy production, FAO induction by leptin leads to elevated production of ATP to fuel cancer cell growth [90]. It was also suggested that leptin shifts the mode of energy production in cancer cells, from glucose consumption to the utilization of fatty acids, thereby favoring the use of glucose for biosynthesis [57]. Second, leptin stimulation of FAO generates a large amount of acetyl-coA molecules, which can be used for post-translational protein acetylation and biosynthesis, in addition to ATP generation [96,97]. Third, through upregulation of FAO, leptin promotes breast cancer stemness and chemoresistance. In this model, mammary adipocyte-derived leptin not only promotes fatty acid uptake from adjacent adipocytes, but also induces FAO to facilitate the self-renewal and chemoresistance of breast cancer stem cells [20]. Mechanistically, leptin induces FAO through JAK/STAT3 signaling pathway-mediated upregulation of carnitine palmitoyl transferase I (CPT-1), an enzyme converting acyl-CoA species to acyl-carnitines for transport into mitochondria, which is a rate-limiting step in long-chain fatty acid oxidation [20,79]. By contrast, activation of PI3K/Akt signaling critically contributes to leptin-modulation of SREBPs and lipogenesis [89]. These different molecular actions provide an interpretation of the relationship between fatty acid synthesis and FAO under leptin stimulation (Figure 2).

**Figure 3:**
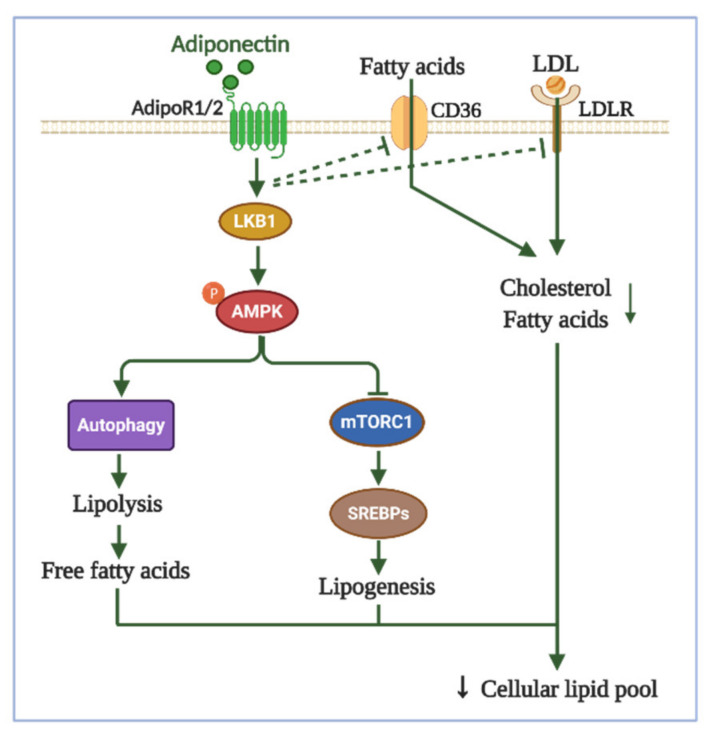
The putative roles of adiponectin in cancer-related lipid metabolic alterations. Adiponectin-driven signaling pathways have been implicated in regulation of lipid metabolism. Adiponectin has been reported to suppress SREBPs-dependent lipogenesis through inactivation of mTORC1, an upstream modulator of SREBPs and de novo lipid biosynthesis. Moreover, adiponectin downregulates expression of CD36 and LDLR, membrane proteins playing an important role in import of exogenous fatty acids and cholesterol, and thereby decreases uptake of lipids from tumor microenvironment. Since adiponectin has been well known as an activator of autophagy, it is speculated that adiponectin induces lipolysis and lipid degradation via β-fatty acid oxidation. Collectively, these effects may lead to impaired reservation of intracellular lipid that in turn possibly causes disruption of membrane structure and interruption of lipid-based signaling pathways. However, the role of adiponectin-promoted lipid metabolic changes remains to be further elucidated. AdipoR1/2: adiponectin receptor 1/2, CD36: cluster of differentiation 36, LDL: low-density lipoprotein, LDLR: the low-density lipoprotein receptor, mTORC1: mammalian target of rapamycin complex 1, SREBPs: sterol regulatory element-binding proteins.

Unlike leptin, the effect of adiponectin on cancer cell-specific lipid metabolism and its contribution to anti-tumor effects of adiponectin are poorly understood. In non-transformed cells, adiponectin has been shown to be a potent inhibitor of lipogenesis. For example, adiponectin suppresses SREBP-1c expression in hepatocytes through activation of the AdipoR1/LKB1/AMPK axis [98]. Although SREBP-1 suppressing effect of adiponectin has not been confirmed in cancer cells yet, this effect is predictable, based on the fact that adiponectin prevents phosphorylation of mTORC1, a key upstream regulator of SREBP-1 and lipid synthesis, via either activation of AMPK or blockage of PI3K/Akt signaling [99]. In cancer-related contexts, mTORC1 inhibition has been also suggested to be responsible, at least in part, for the inhibition of tumor growth by adiponectin [100,101]. In addition, adiponectin phosphorylates acetyl-CoA carboxylase-1 (ACC1), an enzyme that catalyzes the initial and rate-limiting step of de novo fatty acid synthesis, which may further contribute to inhibition of lipogenesis by this adipokine [102]. Along with the putative role of adiponectin as a negative modulator of lipogenesis, there are certain lines of evidence showing that adiponectin is involved in the regulation of lipid uptake in cancer. In human hepatic cancer cells, adiponectin decreases CD36 expression, leading to impaired uptake of palmitic acid [103]. Yet, it remains unclear whether CD36-mediated inhibition of fatty acid uptake contributes to the modulation of tumor growth by adiponectin. Moreover, another study reported that adiponectin suppresses expression of the low-density lipoprotein receptor (LDLR) in mouse primary tumor cells and human breast cancer cells [104]. Consequently, MMTV-PyVT transgenic mice with adiponectin deficiency show enhanced susceptibility to cholesterol-induced mammary tumorigenesis. As an activator of AMPK, adiponectin has been shown to promote FAO in myocytes. Thus, it is possible that adiponectin also affects FAO levels in cancer cells, which may play a role in the multifaceted effects of adiponectin in cancer. However, since many metabolic pathways in cancer cells are far different from those in non-malignant cells, caution should be taken to extrapolate the linkage between modulation of tumor growth and FAO by adiponectin. In fact, metformin, a potent activator of AMPK and FAO under physiological conditions, was demonstrated to inhibit FAO in breast cancer cells [105]. In summary, although adiponectin possesses various effects on lipid metabolism, future investigations are required for unraveling the involvement of adiponectin-induced alterations in cancer-related lipid metabolism in tumor progression. A putative model for adiponectin-driven lipid metabolic alterations is presented in Figure 3.

Recent evidence also suggests the role of other adipokines in cancer cell-specific lipid metabolism. For example, visfatin upregulates fatty acid synthase (FASN), a key enzyme in de novo fatty acid biosynthesis pathway, through Akt- and ERK-dependent mechanisms to promote the growth of prostate cancer cells [71]. Likewise, increased FASN expression was also observed in liver cancer cells exposed to visfatin or resistin [106]. Furthermore, resistin can induce lipid accumulation via upregulation of CD36 [107]. These findings provide initial evidence that visfatin and resistin promote a malignant lipid metabolic phenotype in cancer cells.

### 3.4. Effects of Adipokines on Redox Balance

Reactive oxygen species (ROS), generated as a byproduct of mitochondrial respiration or by enzymatic activity of NADPH oxidases (NOXs), have been believed to play a dual role in tumor growth. On the one hand, low and moderate levels of ROS positively modulate cell proliferation and differentiation, and cellular adaptation to metabolic stress through reverse oxidation of cysteine and/or tyrosine residues on proteins that affects protein function and several signaling pathways [12]. However, excess production of ROS can trigger p38MAPK-dependent apoptosis [108]. In progressive tumors, ROS are overproduced as a consequence of elevated metabolic rate, oncogenic actions, and hypoxia, which requires a powerful antioxidant system for counteracting the lethal effect of excess ROS [109]. Hence, agents that affect cellular ROS levels, either via modulation of ROS production or antioxidant pathways, may regulate the survival and proliferation of cancer cells.

Adipokines have been shown to regulate cellular oxidative stress by controlling redox signaling. However, the effects of individual adipokines on cellular ROS levels are complicated and its role in cancer would be context dependent. Indeed, while leptin promotes NOXs-dependent ROS production in various cell types [110,111], we and other groups have previously shown that the total cellular ROS level remains unchanged or reduces upon long-term treatment with leptin in breast cancer cells [18,112], suggesting a complicated regulatory role of leptin in the modulation of redox response. In fact, leptin has been found to upregulate antioxidant enzymes such as superoxide dismutase (SOD), catalase (CAT), and heme-oxygenase 1 (HO-1), but decreased lipid peroxidase in colorectal and breast cancer cells [113,114]. Furthermore, as previously discussed, leptin diverts glycolytic metabolites into branching pathways for biosynthesis and NADPH production, in which NADPH is a donor of reducing equivalents, essentially required for cellular biosynthetic reactions and maintenance of multiple antioxidant defense systems [12]. For example, leptin increases the activity of the glucose-6-phoshate dehydrogenase (G6PDH) whereby it triggers the pentose-phosphate pathway, a major source of cellular NADPH [57]. In addition, during hypoxia, a trigger of ROS production [115], leptin alleviates oxidative stress by upregulating uncoupling protein 2 (UCP2), which leads to increased uncoupled respiration and decreased mitochondrial ROS generation, signifying that leptin can maintain cellular redox balance to support tumor survival and growth under stress conditions [62]. In summary, current evidence indicates a multifaceted role of leptin in ROS production and redox balance. Leptin may trigger ROS production to promote certain proliferative signaling pathways (e.g., inflammasomes activation [18]) in basal conditions. However, leptin-strengthened antioxidative system prevents ROS-mediated activation of death-inducing pathways in response to metabolic stress.

Similar to leptin, visfatin strengthens the antioxidant defense system by enhancing the activity of antioxidative enzymes, including SOD, CAT, and glutathione peroxidase (GSH-Px), which are implicated in the progression of melanoma [116]. In contrast to the protective role of leptin and visfatin, adiponectin promotes ROS production, leading to redox imbalance and apoptosis in cancer cells. For example, adiponectin increases cellular ROS levels accompanied by induction of autophagy and apoptosis in endometrial cancer cells [117]. Pretreatment of colorectal cancer cells with N-acetylcysteine, a ROS scavenger, abrogates the suppressing effect of adiponectin on cell growth, indicating that anti-cancer effects of adiponectin are mediated partly by inducing oxidative stress [118]. However, it is interesting to note that adiponectin acts as a suppressor of ROS production in non-malignant cells through multiple mechanisms. Compelling evidence has demonstrated that adiponectin reduces NOX2-dependent ROS production, while it increases redox signaling by upregulation of Nrf2/HO-1 axis [119,120,121]. These effects may critically contribute to maintenance of cellular oxidative homeostasis in normal cells, incompliance with the role of adiponectin as a potent suppressor of cancer initiation.

### 3.5. Effects of Adipokines on Other Metabolic Pathways

To date, a large body of evidence supports the view that adiponectin and leptin are critical drivers of glucose and lipid metabolism, whereby they differentially modulate cancer development and progression. Given the recent advances in the identification of new metabolic phenotypes in cancer cells, it will be very interesting to unveil the possible roles of these adipokines in other metabolic pathways related with cancer. A typical example is amino acid metabolism, which has received a growing attention for its critical role in metabolic adaptation of cancer cells. In addition to glucose and fatty acids, amino acids, particularly glutamine and branched-chain amino acids, are also major energy substrates fueling tumor growth [122,123]. More importantly, amino acid metabolism is at the crossroads of biosynthetic pathways of lipids, purines, and pyrimidines [124]. Although little is known about the modulation of amino acid metabolism by adipokines in cancer cells, recent evidence suggests a possibility of crosstalk between adipokines-regulated signaling pathways and amino acid metabolic reprogramming in cancer cells. For example, leptin has been reported to promote tumor growth through upregulation of c-Myc, which is a master regulator of mitochondrial glutaminolysis responsible for glutamine addiction in cancer cells [125,126]. Our preliminary study also suggests that treatment with leptin leads to a significant increase in cellular amino acid levels in breast cancer cells (unpublished data) as a result of elevated uptake or de novo synthesis of amino acids. However, currently the relevant data are not sufficient and further studies are required to gain a better understanding of how amino acid metabolism is remodeled by adipokines.

Cancer development and progression are not only controlled by oncogenic signals in tumor cells, but also by communication between tumor cells and their surrounding stromal cells. In view of the metabolism, cancer cells can reprogram the stroma cells to provide them with essential substrates for energy production and biosynthesis [127]. Indeed, cancer-associated fibroblasts (CAFs), which represent the main stromal cell type in the solid tumor microenvironment, also exhibit the Warburg effect-like metabolic phenotype [128,129]. Reprogrammed CAFs secret a large amount of intermediate metabolites, such as pyruvate and lactate, through aerobic glycolysis into the tumor microenvironment to support tumor growth. While metabolic phenotypes of CAFs have become increasingly recognized, the mechanisms underlying the metabolic alterations in CAFs remain to be fully understood [130]. Interestingly, leptin has been reported to be a mediator in the interaction between tumor cells and CAFs. Accordingly, CAF-derived leptin promotes malignant behaviors of breast cancer cells, in addition to secreting factors, which stimulate reprogramming of adjacent CAFs to produce more leptin and further amplify leptin signaling in cancer cells [131]. Adipokines are also involved in reciprocal regulation of energy metabolism in cancer-associated adipocytes. In the tumor microenvironment, adipocytes show an extensively reprogrammed metabolic phenotype, characterized by increased lipolysis and secretion of leptin, TNF-α, and IL-6, but decreased production of adiponectin [132,133]. Leptin, in combination with adjacent adipocytes-derived fatty acids, trigger FAO to fuel tumor cell proliferation [20,44]. Further insights into the role of adipokines in metabolic linkage between tumor cells and stromal cells would provide novel strategies to reverse tumor-promoting metabolic remodeling.

## 4. Autophagic Regulation: A Potential Mechanism for Cancer Cell Metabolism by Adipokines

Autophagy, a lysosome-mediated degradative process for removing damaged and/or dysfunctional intracellular macromolecules and organelles and recycling cytoplasmic constituents to provide cells with essential metabolic substrates, has been postulated to play a dual role in determining cancer cell fate [134]. While autophagy was originally reported as a distinct type of cell death and certain types of autophagy can induce growth arrest and apoptotic cell death, it has been generally accepted that autophagy supports the survival of tumor cells under stress conditions by supplying recycled metabolites and controlling cellular homeostasis [135]. Autophagy is associated with a wide range of cellular metabolic processes, since activation of this process can generate diverse substrates, including lipids, amino acids, and glucose [136]. Instead, certain types of autophagy regulate specific metabolic pathway. For example, mitophagy controls mitochondrial homeostasis and metabolic function via selective degradation of dysfunctional mitochondria [137]. Likewise, activation of lipophagy leads to the release of free fatty acids from cellular lipid reservoirs [138]. Therefore, autophagy may be an important driver of cancer metabolism and further contribute to metabolic plasticity of cancer cells.

Autophagy induction has been convincingly demonstrated to mediate modulation of tumor growth by adipokines [8]. Interestingly, while both leptin and adiponectin have been shown to activate autophagy, autophagy induction by these adipokines leads to opposite consequences on survival and proliferation of cancer cells. Chung et al. observed that breast cancer cells treated with adiponectin develop a cytotoxic form of autophagy that results in apoptosis induction and enhanced sensitization to chemotherapy [23]. In contrast, autophagy induction by leptin triggers a proliferative signal in estrogen receptor (ER)-positive breast cancer cells [19]. Yet, the mechanisms by which autophagy stimulated by leptin and adiponectin differentially modulates cancer cell fate are poorly understood. Given the critical role of autophagy and leptin/adiponectin in the regulation of cancer metabolism, it could be assumed that autophagy mediates cancer-related metabolic alterations induced by these adipokines. This notion is supported by recent studies showing that autophagy activation is a master modulator of lipid metabolism. In particular, leptin-stimulated autophagy increases intracellular fatty acid availability for FAO to fuel energy production and breast tumor growth [90]. Autophagy is also activated after glutamate depletion and protects cancer cells from impairment of energy production through upregulation of lipid catabolism [139]. In addition, blockage of autophagic flux decreases OXPHOS, leading to lipid accumulation and cell growth arrest in acute myeloid leukemia, revealing an unquestionable linkage between autophagy and FAO levels in cancer cells [140]. It is notable that autophagy activation is also accompanied by increased intracellular lipid reservation in certain contexts. Leptin activation of autophagy is partly responsible for SREBP-1 induction that contributes to elevated lipogenesis in breast cancer cells [90]. Similarly, autophagy stimulation by rapamycin also causes enhanced expression of SREBP-1 in pancreatic cancer cells [141]. These findings strongly suggest that autophagy induction mediates the effects of leptin on lipid metabolic remodeling as discussed in a previous section (4.3). The autophagy-stimulating activity seems to be in line with FAO induction by adiponectin observed in non-transformed cells [102], although it has not yet been confirmed in cancer cells. However, opposite effects of adiponectin and leptin on mTOR activity, other than autophagy induction, may contribute to differential modulation of lipid metabolism by these adipokines.

Mitochondria play a central role in cellular metabolism, which is involved in not only energy production but also biosynthetic pathways and redox balance. Mitophagy has recently emerged as a key factor in the control of mitochondrial health, suggesting that autolysosome-mediated degradation of mitochondria would be a critical mechanism for maintaining malignant metabolic phenotypes [142]. However, defects in mitophagy may also trigger tumor initiation, indicating a dual role of mitophagy in cancer [143,144]. Leptin was reported to stimulate mitophagy to remove damaged mitochondria, which is speculated to mediate enhanced oxidative metabolism by leptin in breast cancer cells [78]. In fact, dysfunctional mitochondria have been linked to a decrease in FAO capacity, as well as oxidation of other substrates, such as glutamine and glucose [145]. Furthermore, given the role of mitochondria as a main source of cellular ROS, leptin-promoted autophagy may contribute to the control of redox balance in cancer cells. Mitophagy activation was also observed upon adiponectin stimulation, which protects myoblasts from apoptosis [121], giving rising to the possibility that mitophagy might drive the metabolic actions of adiponectin in cancer-associated contexts.

Autophagy has been also emerging as a critical regulator of amino acid metabolic rewiring in cancer cells [146]. As mentioned earlier, cancer cells often exhibit glutamine addiction for biosynthesis of ATP and other essential molecules. Due to enhanced dependence on glutaminolysis, tumor cells need to acquire a large amount of glutamine from the surrounding environment through upregulation of glutamine uptake [147,148]. However, since depleted glutamine is commonly observed in tumors, some cancer cells also increase de novo biosynthesis of glutamine to meet the high demand for glutaminolysis [149]. In addition, glutamine recovered from intracellular proteins and organelles through autophagic degradation may be another critical source of glutamine in tumors. Indeed, a recent study demonstrated that blockage of autophagy results in a dramatical reduction in glutaminolysis, which in turn represses gastric cancer growth and metastasis [150]. It is also worthy to note that glutaminolysis negatively regulates autophagy through mTOR activation, suggesting that autophagy and glutaminolysis can mutually modulate to promote cancer metabolism [151,152]. Given the fact that leptin and adiponectin regulate autophagy and mTOR activation, these adipokines may play a multifaceted role in cancer cell-specific glutamine metabolism. Future investigations are needed to provide an insight into the connection between autophagy and amino acid metabolism modulated by adiponectin and leptin in cancer cells.

## 5. Targeting Adipokines-Driven Cancer Metabolism for Counteracting Obesity-Linked Cancer

Given the role of adipokines as modulators of cancer metabolism, targeting adipokines and their downstream signaling molecules can alter tumor metabolic programs and reverse malignant phenotypes of cancer cells. As discussed earlier, adipokines, such as leptin and adiponectin, possess many opposite effects on cellular metabolism. Hence, the imbalance in adipokine production during obesity may facilitate cancer initiation and progression by promoting cancer-cell specific metabolic reprogramming. One logical approach to counteract obesity-related cancer development would be to restore the adipokine signature. Interestingly, while excess adiposity is involved in elevated secretion of leptin and decreased production of adiponectin, intentional weight loss through dietary adjustment and/or exercise has been reported to reverse these changes. Indeed, a large body of preclinical and clinical evidence delineates that weight loss can re-establish the balance in serum adipokine levels [153,154,155]. Although it remains unclear if post-diagnosis weight reduction confers better outcomes in terms of survival in cancer patients, clinical investigations suggest that weight loss decreases serum leptin accompanied by a lower incidence of cancer relapse among survivors with a history of breast cancer and overweight [156,157,158]. In animal studies, energetic interventions by increased physical activity led to reduction in body weight as well as sensitivity of rats to chemical-induced tumorigenesis [159]. Notably, among biomarkers related to cancer development, energetic interventions decreased serum leptin but increased serum adiponectin, suggesting that regulation of adipokine levels by exercise and body-weight control is associated with reduced carcinogenesis.

Some agents that directly interact with receptors of adiponectin and leptin are currently available. For example, a peptide-based leptin receptor antagonist showed to decrease tumor burden in mice bearing orthotopic xenograft breast tumors [160,161]. Likewise, adipoRon, a small-molecule agonist of adiponectin receptors, has been suggested to possess anti-cancer effects in various types of cancer cell [162,163,164]. However, since both leptin and adiponectin exhibit multifaceted roles in cancer metabolism, a wiser therapeutic strategy is to exploit their downstream targets, which control essential metabolic pathways in tumors. One typical example is inhibition of PI3K/Akt, a downstream signaling pathway playing key roles in the effects of leptin on glucose and lipid metabolism. In fact, many pharmacological inhibitors of PI3K/Akt possess potent actions on various metabolic pathways in cancer cells [165,166]. Although several PI3K/Akt inhibitors have been clinically developed for cancer therapy, therapeutic applications of these agents are limited at least in part by their extensive effects on cellular metabolism that cause off-target impacts, such as hyperglycemia, hyperinsulinemia, and reactivation of PI3K as a consequence [167]. Instead, more specific metabolic targets may be aimed to counteract leptin-promoted cancer metabolism. Recently, inhibitors of key enzymes in de novo fatty acid synthesis pathway such as FASN and ACC1/2 have been subjected to clinical trials for cancer therapy [83]. Given that leptin strongly induces fatty acid biosynthesis in cancer cells, these agents may become a promising candidate for treatment of obesity-related cancer.

Taking into consideration that impaired adiponectin signaling is a common consequence of obesity and this adipokine prevents some malignant metabolic phenotypes of tumor cells, adiponectin mimic therapy might be another therapeutic strategy for obesity-linked cancer. It has been suggested that AMPK activators, such as AICAR (5-aminoimidazole-4-carboxamide-1-β-d-ribofuranoside) and metformin, show metabolic effects similar to adiponectin under physiological conditions [168,169,170]. Furthermore, compelling evidence indicates that AMPK activators suppress tumor growth though inhibition of de novo lipogenesis [105,171,172]. Therefore, reactivation of AMPK may serve as a potential intervention to reverse promoting effects of obesity on cancer metabolism.

## 6. Conclusions and Perspectives

Mounting evidence has shown that adipokines, in particular leptin and adiponectin, modulate cancer cell-specific metabolism in a complicated manner. Leptin promotes glycolysis, a typical metabolic phenotype of tumors, but also supports mitochondrial respiration through remodeling of lipid metabolism and FAO induction, thus promoting the survival and proliferation of cancer cells. While many aspects of adiponectin-modulation of cancer metabolism need to be further elucidated, adiponectin appears to be a negative modulator of the Warburg effect and de novo lipogenesis that may be responsible for its tumor suppressing effects. The multifactorial regulation of leptin and adiponectin in tumor metabolic reprogramming is mediated via extensive effects of these adipokines on key signaling pathways that control cellular metabolism, including activation of PI3K/Akt/mTOR and JAK/STAT by leptin, and modulation of AMPK/mTOR by adiponectin. In addition, autophagy is another critical mechanism underlying differential metabolic functions of adiponectin and leptin.

Although metabolic activities of adipokines have been demonstrated to critically contribute to various obesity-linked complications, the direct evidence on the linkage between many altered metabolic pathways and modulation of tumor growth by adipokines is lacking. Given that obesity differentially modulates the secretion of adipokines, and this could be implicated in obesity-induced cancer, further insights into the role of adipokine-driven metabolic reprogramming in cancer development and progression would provide better therapeutic strategies to counteract obesity-promoted cancer.

## Data Availability

No new data were created in this study. Data sharing is not applicable for this article.

## Abbreviations

CAFs	Cancer-associated fibroblasts
FAO	Fatty acid β-oxidation
ObR	The leptin receptor
OXPHOS	Oxidative phosphorylation
ROS	Reactive oxygen species
TCA	Tricarboxylic acid cycle
